# Evaluation of the Efficacy of Xyloglucan, Pea Protein and *Opuntia ficus-indica* Extract in a Preclinical Model of Psoriasis

**DOI:** 10.3390/ijms24043122

**Published:** 2023-02-04

**Authors:** Alessia Filippone, Giovanna Casili, Marika Lanza, Sarah Adriana Scuderi, Alessio Ardizzone, Anna Paola Capra, Irene Paterniti, Michela Campolo, Salvatore Cuzzocrea, Emanuela Esposito

**Affiliations:** Department of Chemical, Biological, Pharmaceutical and Environmental Sciences, University of Messina, 98166 Messina, Italy

**Keywords:** psoriasis, *Opuntia ficus-indica* extract, xyloglucan, pea protein, barrier function

## Abstract

Psoriasis is a chronic inflammatory skin disease characterized by epidermal gene abnormalities, epidermal barrier defects and inflammation. Corticosteroids are considered to be standard treatments, but often come with side effects and lose efficacy with long-term use. Alternative treatments targeting the epidermal barrier defect are needed to manage the disease. Film-forming substances such as xyloglucan, pea protein and *Opuntia ficus-indica* extract (XPO) have generated interest for their ability to restore skin barrier integrity and may pose an alternative approach to disease management. Thus, the aim of this two-part study was to evaluate the barrier-protective properties of a topical cream containing XPO on the membrane permeability of keratinocytes exposed to inflammatory conditions and compare its efficacy to dexamethasone (DXM) in an in vivo model of psoriasis-like dermatitis. XPO treatment significantly reduced *S. aureus* adhesion, subsequent skin invasion and restored epithelial barrier function in keratinocytes. Furthermore, the treatment restored the integrity of keratinocytes, reducing tissue damage. In mice with psoriasis-like dermatitis, XPO significantly reduced erythema, inflammatory markers and epidermal thickening with a superior efficacy to dexamethasone. Given the promising results, XPO may represent a novel steroid-sparing therapeutic for epidermal-related diseases such as psoriasis, thanks to its ability to preserve skin barrier function and integrity.

## 1. Introduction

Psoriasis is a chronic inflammatory skin disease characterized by hyperproliferation and abnormal differentiation of epidermal keratinocytes, infiltration of inflammatory cells and hyperplasia of dilated superficial dermal vessels [1]. Psoriasis affects people of all ages and in different geographic areas; the prevalence in countries ranges between 0.09% and 11.43%, making psoriasis a serious global health burden with at least 100 million individuals affected worldwide [2]. The pathophysiology of psoriasis comprises epidermal abnormalities including barrier defects, abnormal hyperproliferation and differentiation of keratinocytes, infiltration of T cells and neutrophils, leading to inflammation and erythema [1]. It has been demonstrated that cell death of keratinocytes and barrier defects result as a subsequent T cell-targeted process in the epidermis of human psoriatic lesions, where CD4^+^ T and CD8^+^ T cell activation, together with a release of inflammatory cytokines and chemokines, drive the pathogenesis of psoriasis [3]. Increasing scientific evidence supports skin barrier abnormality as an underlying pathogenesis of psoriasis [4]. Under physiological conditions, the cell growth cycle lasts 28–30 days; however, in psoriasis, skin cells develop faster (within 3–10 days) and migrate from the basal layer to the stratum corneum, leading to an excessive deposition of immature cells [5]. Topical corticosteroids (CS) represent the most commonly prescribed treatment for psoriasis, with dexamethasone (DXM) being one of the recommended treatments. Despite the known efficacy of conventional therapies to decrease signs and symptoms severity, side effects may appear, such as atrophy, development of striae, steroid rosacea and skin bacterial superinfections [6]. Indeed, novel therapeutical strategies for psoriasis treatment could be represented by alternative approaches. In recent years, naturally derived ingredients such as polysaccharides [6,7], extracts [8] and essential oils [9] have gained much attention for their health benefits and tolerability. Tamarind seed gum (Xyloglucan) is a hemicellulose extracted from the seed of the tamarind tree, Tamarindus indica. It is a non-ionic, neutral, branched polysaccharide that consists of a cellulose-like backbone carrying xylose and galactoxylose, present in the primary cell walls of all vascular plants [10]. Xyloglucan is characterized by a structural similarity to mucin and it is able to exert a protective barrier effect on mucosal and skin surfaces which restores the integrity of mucosal cells, protecting them from damaging agents, such as microorganisms and allergens [11,12]. In an in vivo model of atopic dermatitis (AD) and gastroenteritis associated with urinary tract infections (UTIs), xyloglucan was shown to possess protective barrier effects counteracting histological damage and helping to reduce inflammation [13,14]. Similar protective effects have been reported for pea protein extracted from peas (*Pisum sativum* L.) that helps promote tissue repair, supports epithelial barrier integrity and helps reduce inflammatory symptoms in mice with dextran sulfate sodium (DSS)-induced colitis [15]. Moreover, protective and antioxidant activities have been reported for *Opuntia ficus-indica* (OFI) extract (belonging to the Cactaceae family), containing a mixture of mucilage and pectin that can stimulate mucus production, forming a protective layer that, together with its water-holding capacity, moisturizes the skin [16]. Therefore, the aim of this study was to evaluate the barrier-protective properties of a topical product containing xyloglucan, pea protein and *Opuntia ficus-indica* extract (XPO) in an in vitro model of psoriasis utilizing a layer of keratinocytes stimulated by a cocktail of cytokines and *S. aureus* infection, and in an in vivo model of IMQ-induced psoriasis [17,18].

## 2. Results

### 2.1. Effect of XPO on Skin Barrier Function Assessed by Trans-Epithelial Electrical Resistance (TEER) Test

**Figure 1:**
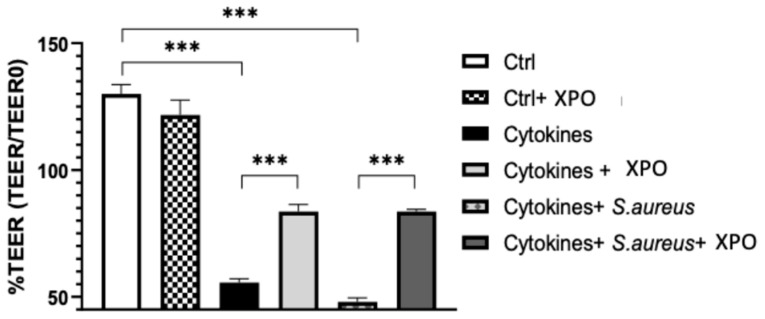
TEER measurement. The barrier-protective properties of XPO on keratinocytes were assessed through the TEER test, observing that HaCaT stimulation with cytokines mix and cytokines mix + *S. aureus* significantly reduced TEER measurement compared to control cells; interestingly, XPO treatment prevented TEER decrease, highlighting the role of XPO in supporting epithelial barrier integrity in keratinocytes. Values are indicated as the mean ± SEM. *** *p* < 0.001.

Non-invasive techniques are best suited to continuously monitor skin barrier integrity, including TEER, which represents a sensitive and reliable method to confirm the integrity and permeability of a cellular monolayer [19]. To evaluate the barrier-protective properties of XPO on keratinocytes, we assessed barrier structure and epithelial thickness with TEER, observing that HaCaT stimulation with cytokines mix and cytokines mix + *S. aureus* significantly reduced TEER measurement compared to control cells (Figure 1); interestingly, XPO treatment prevented TEER reduction, highlighting the ability of XPO to support epithelial barrier integrity in keratinocytes (Figure 1). 

### 2.2. Role of XPO in Modulating Keratinocyte Membrane Integrity with Lucifer Yellow (LY) Permeation Test

**Figure 2:**
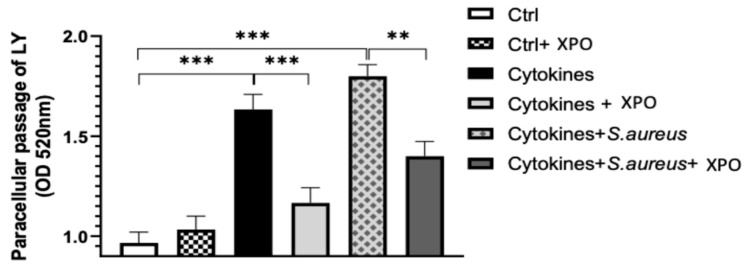
LY permeation test. LY assay showed that stimulation with cytokines mix or with cytokines mix + *S. aureus* after 24 h significantly increased the paracellular flux of LY compared to unstimulated HaCaT cells, while XPO reduced LY permeation, indicating reduced epithelial permeability. Values are indicated as the mean ± SEM. *** *p* < 0.001 and ** *p* < 0.01.

To evaluate the protective properties of XPO on keratinocyte membrane integrity and permeability, an LY assay was used. Here, the fluorescent hydrophilic molecule can pass across the epidermal cells through passive paracellular diffusion, thus acting as a marker of membrane integrity. Markers of membrane integrity include tight junction proteins, intercellular contacts that make up the physical skin barrier and regulate epithelial permeability [20]. Stimulation with cytokines mix or with cytokines mix + *S. aureus* after 24 h significantly increased the paracellular flux detected by LY compared to unstimulated HaCaT cells (Figure 2), while the treatment with XPO reduced LY permeation across the monolayer, suggesting an important role in maintaining membrane integrity and permeability (Figure 2).

### 2.3. Effects of XPO Treatment on Cell Damage through Lactate Dehydrogenase (LDH) Assay

**Figure 3:**
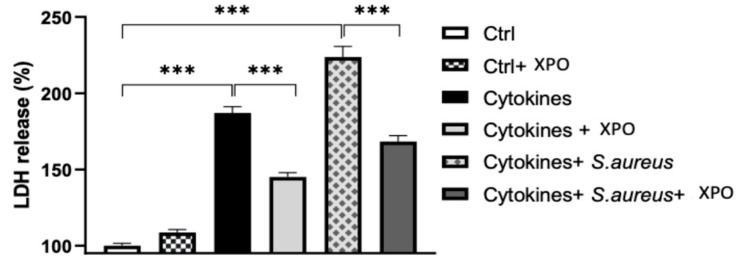
LDH release. LDH release was significantly increased in the cell culture supernatant after incubation with cytokines or with cytokines + *S. aureus* compared with control group. XPO treatment reduced the percentage of LDH released by HaCaT keratinocytes. Values are indicated as the mean ± SEM. *** *p* < 0.001.

During the onset of psoriasis, the alteration of cellular physiology leads to the activation of programmed cell death [21]. The harmful effect of stimulating cells with bacteria and cytokines was determined by performing an LDH assay to evaluate the barrier-protective properties of XPO in preventing keratinocyte damage. LDH release was significantly increased in the cell culture supernatant after incubation with cytokines and cytokines + *S. aureus* compared with the control group (Figure 3). The treatment with XPO was able to counteract the damaging effects of both stimulations with cytokines mix only and cytokines mix + *S. aureus*, reducing the percentage of LDH released by HaCaT keratinocytes, as seen in Figure 3. These data highlight the capacity of XPO treatment to significantly counteract cell damage induced by pro-inflammatory cytokines and *S. aureus* infection.

### 2.4. Role of XPO Treatment to Counteract Bacterial Adhesion and Invasion in Differentiated HaCaT Cells

**Figure 4:**
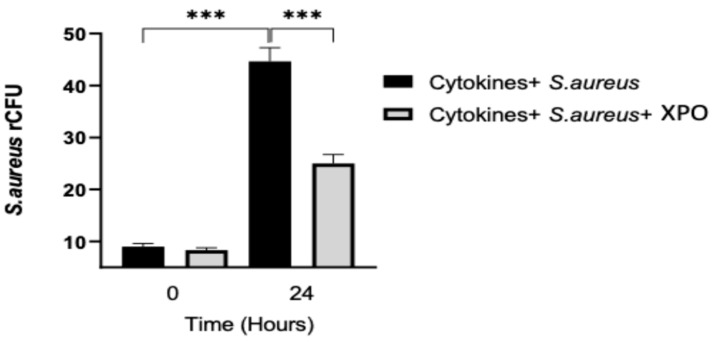
Relative number of CFUs (rCFUs). *S. aureus* adhesion rate was calculated and the relative number of CFUs (rCFUs) was estimated by plating out the lysate of infected cells and counting the number of CFUs at T0 and T24 h. XPO treatment was able to prevent *S. aureus* infection, reducing bacterial adherence. Values are indicated as the mean ± SEM. *** *p* < 0.001.

*S. aureus* is a commensal organism and a frequent cause of skin and soft tissue infections, which can progress to serious invasive diseases. In fact, the skin microbiome of psoriasis patients is reported to have an elevated presence of Staphylococcus genus [22]. To evaluate the capacity of XPO to protect HaCaT cells from bacterial adhesion and invasion, the *S. aureus* adhesion rate was calculated and the relative number of colony-forming units (CFUs) was estimated by plating out the lysate of infected keratinocytes and counting the number of CFUs at each time point (T0 and T24 h). As shown in Figure 4, XPO treatment was able to reduce bacterial adherence. Thus, XPO demonstrated its ability to significantly prevent *S. aureus* infection by avoiding its adhesion and invasion on epidermal keratinocytes.

### 2.5. Effects of XPO Cream on Histological Changes in Mice with IMQ-Induced Psoriasis Like-Dermatitis

**Figure 5:**
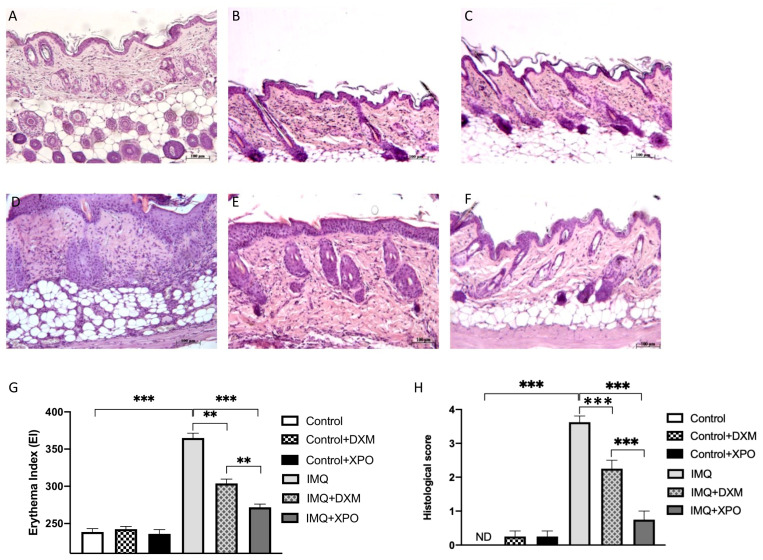
Effects of XPO cream on histological damage induced by imiquimod (IMQ) and erythema evaluation. Extensive damage to the epidermis was evaluated in IMQ-treated mice (panel (**D**)). Administration of DXM reduced tissue damage caused by IMQ (panel (**E**)) compared to the control group (panel (**A**). Topical treatment of DXM (**C**); Topical treatment of XPO (**B**); Topical treatment of XPO cream significantly reduced histological damage induced by IMQ (panel (**F**)); see histological score (**H**). IMQ treatment increased erythema and the thickness of skin in psoriasis-like mouse model compared to control group (**G**). However, mice treated with XPO cream by topical administration revealed markedly reduced erythema in the lesioned flank, with higher efficacy compared to DXM (**G**). *** *p* < 0.001; ** *p* < 0.01; ND: not detectable.

In psoriasis, the skin layer architecture is dramatically modified, with severe dermal inflammation and hyperkeratosis worsening the outcome; DXM represents one of the conventional topical treatments for psoriasis [23]. To evaluate the ability of XPO cream in reducing erythema in mice with psoriasis-like dermatitis, histological analysis and erythema index were performed. We found that IMQ-treated mice showed noticeable erythema, characterized by redness of the inflamed skin, flaky scaled skin and increased skin thickness compared to the control group (Figure 5G). XPO significantly reduced erythema in the injured flank and epidermal thickening with improved efficacy compared to mice treated with DXM cream (Figure 5G). Moreover, to evaluate alterations of the skin architecture in mice with psoriasis-like dermatitis, H&E staining was performed. We observed that IMQ-treated mice showed extensive damage to the epidermis, thickening of the stratum corneum (known as a hyperkeratosis) and infiltration of immune cells (Figure 5D, see histological score H). Notable clinical signs of psoriasis linked to an alteration of the epidermal barrier integrity [24] compared to the control group (Figure 5A, see histological score 5 H) were also observed. In fact, the data showed a significant reduction in tissue damage and dermal neutrophils infiltration after damage induced by the daily application of IMQ (Figure 5E, see histological score H). Topical administration of XPO cream (Figure 5F, see histological score H) significantly reduced tissue damage, better than the DXM treatment alone. These results demonstrated that the topical treatment with XPO cream can effectively improve skin architecture and reduce histological tissue damage in mice with psoriasis-like dermatitis, creating a protective mechanical layer able to restore barrier integrity.

### 2.6. Protective Effect of XPO Cream on Mast Cell Degranulation and Spleen Weight

**Figure 6:**
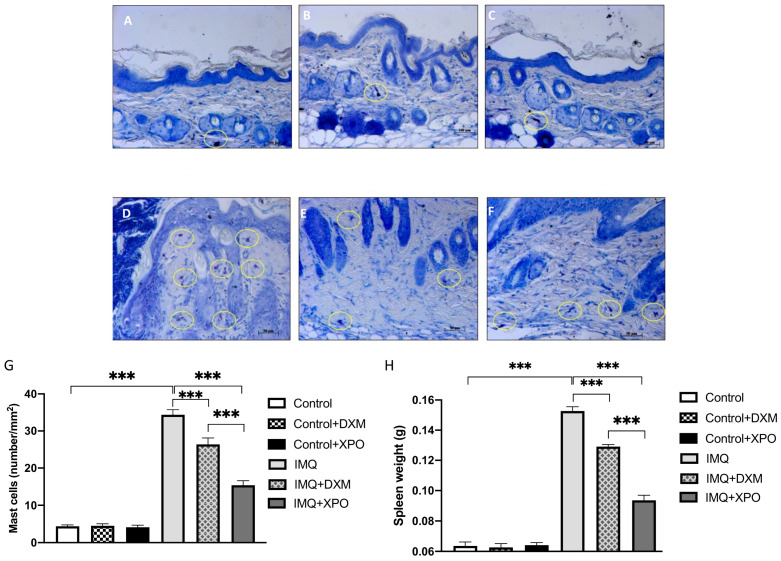
Effect of XPO cream on mast cell infiltration induced by imiquimod (IMQ) and spleen weight. Mice treated with IMQ displayed a significant increase in mast cell degranulation which was determined by toluidine blue staining (panels (**A**–**F**)). XPO cream treatment significantly reduced the degranulation of mast cells (panel (**F**)). See mast cell counts (**G**). Marked splenomegaly was diagnosed in the psoriatic mice (**H**), while a marked reduction in this systemic effect was evident following XPO treatment (**H**). *** *p* < 0.001.

Mast cells are involved in the inflammatory process of psoriasis, especially in the early pathological stages [25]; therefore, the efficacy of XPO cream in reducing mast cell degranulation in mice with psoriasis-like dermatitis was evaluated. Our data showed that control mice did not report a relevant number of mast cells (Figure 6A–C, see densitometric analysis G), while IMQ-treated mice showed an evident increase in mast cell numbers (Figure 6D, see densitometric analysis G). XPO topical administration was able to significantly reduce mast cell degranulation (Figure 6E, see densitometric analysis 6 G) compared to DXM treatment alone (Figure 6F, see densitometric analysis 6 G). Moreover, in autoimmune disorders such as psoriasis, the altered barrier is associated with continuous activation of the immune system, which maintains a state of constant inflammation occurring mainly in the secondary lymphoid organ [26]. In fact, a known feature of the IMQ psoriasis-like mouse model is the splenomegaly, an endpoint often addressed to identify potential effective therapeutic candidates for the treatment of this pathology [27]. IMQ-treated rodents displayed marked splenomegaly compared to the normal spleen weight of the control mice. DXM treatment of psoriatic mice significantly reduced the size of the spleen, and this reduction was even more accentuated following XPO cream therapy in IMQ-treated mice, confirming the efficacy of XPO treatment in helping to reduce inflammation in mice with psoriasis-like dermatitis (Figure 6H). 

## 3. Discussion

Psoriatic patients are often subjected to bacterial infections that exacerbate the disease, particularly due to Staphylococcus and Streptococcus strains; *S. aureus* is considered the most prevalent bacterium in patients with superinfected psoriasis, with a frequency of 46% [28]. Despite the evolving understanding of its pathogenesis, no lasting cure has been found that can specifically reduce skin infections while managing the symptoms. The topical therapeutical approach for plaque psoriasis mainly involves the use of topical corticosteroids and vitamin D3 analogues, but despite clear benefits, these come with a variety of side effects [29]. Thus, novel therapeutical compounds represent an innovative alternative to conventional drugs that should be considered. To date, plant-derived compounds have been demonstrated to play important roles as alternative anti-psoriasis agents [30]. Natural polysaccharides such as XG possess a molecular structure that confers mucoadhesive properties to various epithelial tissues, allowing them to counteract bacterial adherence and invasion and to preserve tight junctions and paracellular flux, as evidenced in different in vitro and in vivo studies [31,32,33]. An emerging protective role is played by PP, which is able to create a protective synergistic mechanical layer on epithelial tissues together with XG thanks to its gelling properties [13]. Lastly, *Opuntia ficus-indica* extract possesses an antioxidant effect [34], is rich in mucilage and has been reported to have a beneficial outcome on wound healing activity [35]. Given the well-known properties of xyloglucan, pea protein and *Opuntia ficus-indica* extract, we aimed to investigate the combined barrier-protective effect of a unique cream mixture (XPO) on epidermal damage in an in vitro model of psoriasis through cytokine stimulation and *S. aureus* infection, and in an in vivo model of psoriasis like-dermatitis induced by IMQ. The skin is the largest organ of the human body and accomplishes multiple defensive and regulatory functions. The skin barrier function resides in the epidermis, mainly in the stratum corneum [30,36]; this epidermal barrier maintains cutaneous homeostasis and protects the body against numerous external stressors [36,37]. Keratinocytes are the major cell type in the skin and provide an effective barrier that protects against the invasion of microorganisms and various antigens; particularly, *S. aureus*, one of the most important human pathogens that causes various superficial and systemic infections, has been implicated as a causative or exacerbating agent in a broad range of skin diseases, including psoriasis [37]. Pathological mechanisms, intervening in psoriasis, can alter skin barrier properties, modifying the proliferation process of the corneocytes [21,30]. The loss of this equilibrium leads to an impaired function of the skin barrier [36,38]. In the in vitro study, the evaluation of TEER, a sensitive and reliable method to confirm the integrity and permeability of the monolayer, demonstrated that the integrity of HaCaT cells was strikingly compromised by inflammatory cytokines and *S. aureus* infection, with XPO significantly restoring permeability and integrity. The LY assay is a robust paracellular permeability marker to evaluate monolayer integrity [20,30]. This study demonstrated the beneficial properties of XPO treatment to reduce LY permeation across the monolayer. This was of particular interest because it reduces the increased paracellular permeability caused by cytokines and/or bacterial infection. Psoriatic lesions are maintained by the complex interplay between T cells and their cytokines, vascular endothelium and epidermal keratinocytes that can in turn stimulate keratinocytes. This forms an amplification feedback loop for the inflammatory reaction, which can compromise skin cell membranes [39,40]. An important indicator of cell membrane integrity is represented by LDH release. When the cell membrane is disrupted, LDH, an intracellular enzyme, is released from the damaged cells [41]. In our study, the LDH content in the medium of stimulated or infected HaCaT cells, treated with XPO, was significantly decreased compared to non-treated cells, highlighting the protective action mediated by XG, PP and OFI to preserve cells from cellular damage. The ability of *S. aureus* to survive in keratinocytes after internalization contributes to the development of persistent or chronic infections, allowing the bacteria to disseminate deeper into the tissue [18]. Here, the number of internalized bacterial colonies drastically increased within 24 h in infected keratinocytes, while XPO treatment reduced bacterial adherence to keratinocyte membranes, slowing down *S. aureus* colonization, most likely due to the mucomimetic mechanical barrier formation of XPO. In addition, we decided to evaluate the effect of the product XPO in a murine model of psoriasis to confirm its efficacy in reducing tissue damage and symptoms in vivo. In our murine model of psoriasis like-dermatitis, we found that IMQ treatment up to day 7 led to a marked increase in erythema, thickness in the stratum corneum and induced tissue exacerbations such as skin inflammation, keratinocyte hyperproliferation and leukocyte infiltration. Topical administration twice a day of both XPO cream and DXM led to reduced erythema and epidermal thickening. No direct damage was reported in the skin of control mice treated with XPO or DXM alone, confirming their safety. The defective skin barrier and keratinocyte hyperproliferation allows for activated resident immune cells and mast cells to accumulate, key players that drive psoriasis. We observed an increase in mast cells in the IMQ-treated mice, which is consistent with the increase seen in previous studies [42], as IMQ also acts as a chemoattractant for mast cells and leads to their degranulation. Conversely, compared to the IMQ group, mast cell degranulation in psoriatic lesions was significantly reduced by XPO administration, suggesting that the treatment is able to maintain skin homeostasis with greater efficacy compared to DXM. Moreover, after 7 days, the spleen size in IMQ-treated mice had increased due to the deregulated inflammatory immune reaction during psoriasis, whereas XPO topical administration significantly decreased spleen size, avoiding the severity of inflammatory immune activation. Furthermore, XPO was able to reduce the size of the spleen with increased efficacy compared to DXM treatment. Our data suggest that xyloglucan, pea protein and *Opuntia ficus-indica* extract are able to synergistically create a mechanical protective barrier that restores the physiological skin barrier function, preventing abnormal proliferation of keratinocytes and therefore avoiding psoriatic plaque formation. The IMQ-induced skin inflammation mouse model is currently an accepted psoriasis model because it reports many of the significant consequences observed in human disease, including stratum corneum lesions, specific cytokine expression and cellular infiltrates. Nevertheless, our study does come with a set of limitations, because despite the research advancements made thus far on the behavior of skin cells in psoriasis, currently, no in vitro model perfectly reproducing the disease exists. Firstly, in vitro models using cytokine stimulation are often used to test the efficacy of topical and/or systemic pharmaceutical compounds rather than naturally derived substances for the treatment of psoriasis. In addition, their usage is limited in practice, due to the rather poor histological characterization, the absence of blood vessels, the presence of an unspecific microenvironment, the complete lack of information about possible hyperproliferation of KC and/or the absence of a dermal part [43]. Therefore, in the future, MatTek’s EpiDerm model [44] could be used to understand XPO’s mechanism of action. MatTek’s EpiDerm is an in vitro psoriatic skin model better suited to characterize the presence of acanthosis, a condition linked to hyperkeratosis of the skin, which is a key feature of psoriasis. Furthermore, it would be of interest to investigate the effect of XPO in preventing the adhesion of other bacterial strains, as Firmicutes, Actinobacteria and Proteobacterium, that play a role in the exacerbation of psoriasis [45]. Future perspectives could be directed to an accurate clinical investigation of the potential properties of XPO cream in helping to re-establish the skin permeability and integrity recovery in order to suppress epidermal scaling, which can further exacerbate disease progression. In this context, the physiological changes in the skin barrier function in psoriasis could be evaluated by measuring the extent of trans-epidermal water loss (TEWL) [46]. Despite these limitations, our results clearly demonstrate that skin barrier dysfunction can be managed by the topical application of XPO in psoriasis, which would reduce the use of topical corticosteroids and the negative side-effects that come with them, although future experiments are needed to demonstrate whether the XPO efficacy is translational to the treatment of human psoriasis. Indeed, natural compounds might pave the way for potential new safe and effective approaches to manage psoriasis as well as other skin-related diseases. 

## 4. Materials and Methods

### 4.1. In Vitro Study

#### 4.1.1. Cell Line

In this study, HaCaT cells were used. Cells were purchased from ATCC (PCS-200-011™). HaCaT cells are a spontaneously immortalized, human keratinocyte line that has been widely used for studies of skin diseases [47]. HaCaT cells are round and display a high mitotic index. At near 80% confluence, the cells form colonies. These cells were cultured in complete growth medium consisting of Bovine Pituitary Extract (BPE) 0.4%, rh TGF-a 0.5 ng/mL, L-Glutamine 6 mM, Hydrocortisone Hemisuccinate 100 ng/mL, rh Insulin 5 mg/mL, Epinephrine 1.0 mM and Apo-Transferrin 5 mg/mL.

#### 4.1.2. *S. aureus* Culture

Bacteria were grown in caso-bouillon at 37 °C with gentle shaking for 24 h. The bacteria were then harvested by centrifugation and the pellet was resuspended in Stemline Keratinocyte Medium (SKM). The bacterial density was adjusted to 108 CFU/mL and used as a stock solution. Serial dilutions of this concentration were prepared as previously described [48].

#### 4.1.3. In Vitro Model of Psoriasis

HaCaT cells were cultured in 40 mm Petri dishes with Stemline Keratinocyte Medium (SKM), completed with Fetal Calf Serum (FCS) 5% for 72 h to induce psoriatic differentiation. When cells reached the confluence of 80%, the medium was supplied with a mix of pro-inflammatory cytokines—IL-1α (10 ng/mL) (Peprotech, London, UK; cat#200-01A-10UG), TNF-α (5 ng/mL) (Millipore, Burlington, MA, USA; cat#GF023) and IL-17A (10 ng/mL) (Peprotech, London, UK; cat# 200-17-25UG)—for 24 h [43,49], as inflammatory cytokines are involved in the progress of psoriasis and lead to functional defects of the epidermal barrier [17]. Particularly, the combination of proinflammatory cytokines (IL-17A, IL-1α and TNF-α) provoked keratinocytes to secrete chemokines and antimicrobial peptides with increased proliferation and decreased differentiation, recapitulating key features of psoriasis [50].

#### 4.1.4. *S. aureus* Infection

Skin colonization by *S. aureus* promotes the severity of skin lesions [17]. To induce infection post-differentiation, cells were inoculated with *S. aureus* containing approximately 5 × 107 CFU suspended in DMEM. This bacterial inoculum was added to each well, and the plates were incubated at 37 °C in a 5% CO2-95% air atmosphere for 90 min. Nonadherent bacteria were held off; then, cells were treated with cytokines mix for 24 h [18].

#### 4.1.5. Experimental Groups

Group 1: CON (keratinocytes, KC). After differentiation, complete medium was added to cells for 24 h.

Group 2: CON + XPO. After differentiation, complete medium + XPO (0.1 g/mL) was added for 24 h.

Group 3: Cytokines mix (psoriasis model). After differentiation, the cells were treated with cytokines mix for 24 h.

Group 4: Cytokines mix + XPO. After differentiation, the cells were treated with XPO (0.1 g/mL) for 2 h and then exposed to cytokines mix.

Group 5: Cytokines mix + *S. aureus*. After differentiation, the cells were infected with *S. aureus* for 90 min and then exposed to cytokines mix for 24 h.

Group 6: Cytokines mix + *S. aureus* + XPO. After differentiation, the cells were treated with XPO (0.1 g/mL), infected with *S. aureus* and then exposed to cytokines mix for 24 h. 

#### 4.1.6. TEER Measurement

The barrier integrity was assessed with TEER, a quantitative measure of electrical ohmic resistance of the cell layer. For the TEER method, STX2 electrodes and a 4-point measurement system on Transwell permeable inserts were used [51]. To measure TEER, two sets of electrodes were used; one was placed in the basolateral compartment and the other was placed in the apical compartment [52]. Cells were seeded at a high plating density (1.25 × 105 cells/cm^2^) on clear polyester Transwell permeable supports (Corning Glass) in growth medium. Growth medium (apical and basal) was changed every day before and during treatments. When TEER reached a plateau (2–3 weeks after seeding, ∼3000 ohms·cm^2^), XPO 5% was added to the culture medium. An EVOM epithelial volt/ohmmeter with STX (chopstick) electrodes (World Precision Instruments, Sarasota, FL, USA) was used to measure TEER.

#### 4.1.7. LY Permeation

LY is a fluorescent dye used as a marker of membrane integrity. For this assay, cells were plated into 96-well plates with polycarbonate membrane inserts at a seeding density of 50,000 cells/cm2. When cells reached confluency on the 5th day, they were treated with dilutions of XPO 5%. Subsequently, the cells were infected with *S. aureus* (SA). On the 6th day, the monolayer integrity was evaluated by measuring the LY permeation across the barrier, as previously described [53]. To initiate the LY transport experiment, 50 µL of transport buffer (141 mM NaCl, 4 mM KCl, 2.8 mM CaCl2, 1 mM MgSO4, 10 mM HEPES and 10 mM D-glucose, pH 7.4) containing 100 µM LY was loaded into the apical side of each Transwell filter; 200 µL of transport buffer was added to the basolateral side of each Transwell filter in a 96-well plate (Corning Inc., Corning, NY, USA). The Transwell plate was then placed in a 37 °C, 5% CO2 incubator for 1 h. The LY concentrations in the apical and basolateral compartments were determined by measuring their fluorescence intensities compared to the standard curves of LY at excitation and emission wavelengths of 485 and 529 nm, respectively, using a microplate reader (Biotek, Winooski, VT, USA).

#### 4.1.8. LDH Assay

The measurement of the LDH activity present in the culture supernatant was used as a marker of membrane integrity of the keratinocytes, as previously described [54]. For this evaluation, a CytoTox 96 nonradioactive cytotoxicity assay was used (Promega, Madison, WI), following the manufacturer’s instructions. Maximum release was achieved by lysing the monolayers with Triton X-100 at a final concentration of 1% (*v*/*v*). The activity of the enzymes was determined using a microplate reader (determination of the absorbance at 492 nm). Membrane integrity was calculated as follows: percent integrity = (amount of LDH released in the test − amount released spontaneously) / (maximal amount released − amount released spontaneously).

#### 4.1.9. rCFU Evaluation

The cells were washed 3 times with PBS and then incubated for 15 min at 37 °C with 0.02% Triton X-100 to lyse cells and release the intracellular bacteria. Various dilutions of the lysates were plated on BHI agar plates and incubated overnight at 37 °C. The keratinocytes were lysed by PBS. The adhesion rate was calculated as follows: CFU of adhered bacteria/CFU of the initial number of bacteria.

### 4.2. In Vivo Model of Psoriasis

#### 4.2.1. Animals

Six-week-old male BALB/c mice (25–30 g, Envigo, Udine, Italy) were properly housed in a controlled environment (22 ± 2 °C, 55% ± 15% relative humidity, 12 h light/dark cycle) and steel cages were periodically cleaned. The animals were provided with food and water ad libitum. This study was approved by the University of Messina Review Board for the care of animals, in compliance with Italian regulations on the protection of animals (no. 549/2018-PR released on 23 February 2018). Animal experiments followed Italian regulations on the protection of animals used for experimental and other scientific purposes (D. Lgs 2014/26 and EU Directive 2010/63).

#### 4.2.2. IMQ-Induced Psoriasis-like Dermatitis Murine Model and Treatments

An imiquimod (IMQ)-induced psoriasis-like dermatitis murine model was induced by topical administration of IMQ cream (62.5 mg/day for 7 days) on the right flank skin (1.5 × 1.5 cm area) that had been previously depilated. XPO and DXM were topically applied (daily for 5 days) 4 h after IMQ application. Control mice were treated with Vaseline cream. The used compounds were dissolved in a cream of vegetable origin. 

#### 4.2.3. Experimental Groups

Mice were randomly divided into the following groups:

Group 1: Control group. Mice received vehicle (Vaseline cream) without IMQ treatment for 5 days (*n* = 4).

Group 2: Control + DXM group. Mice received DXM cream without IMQ treatment for 5 days (*n* = 8).

Group 3: Control + XPO. Mice received XPO cream without IMQ treatment for 5 days (*n* = 8).

Group 4: IMQ group. Mice received vehicle with IMQ treatment for 5 days (*n* = 8);

Group 5: IMQ + DXM group. Mice received DXM cream with IMQ treatment for 5 days (*n* = 8).

Group 6: IMQ + XPO. Mice received XPO cream with IMQ treatment for 5 days (*n*= 8).

All of the experimental groups were sacrificed on day 7. Erythema scoring was performed on day 7. After sacrifice, the spleens were removed, collected and weighed, and right flank skin was collected for further analysis.

#### 4.2.4. Histological Examination

After fixing the skin tissues in buffered formaldehyde solution (4% in phosphate buffered saline (PBS)), sections (7 µm) were prepared and stained with hematoxylin and eosin (H&E) as previously described [55] and then observed under a light microscope (Leica QWin V3, Cambridge, United Kingdom). Histological damage was assessed using a score from 0 to 4. To be precise, the erythema, scaling and thickening were scored independently on a scale from 0 to 4 (0, none; 1, slight; 2, moderate; 3, marked; 4, very marked).

#### 4.2.5. Toluidine Blue Staining

Cell quantification of mast cells was performed using toluidine blue dye for 4 min. After dehydration in graded ethanol and xylene, the sections were fixed on glass slides using Eukitt (Bio-Optica, Milan, Italy). The number of metachromatic stained mast cells was obtained by counting in five high-power fields (40×) per section by using an Axiovision Zeiss (Milan, Italy) microscope-correlated software (Carl Zeiss Vision, Jena, Germany) [55]. 

#### 4.2.6. Spleen Weight

On day 7, the spleens were collected, dried on filter paper and immediately weighed [26].

### 4.3. Materials

All compounds used in vitro and in vivo were purchased from Sigma-Aldrich Company Ltd. (Poole, United Kingdom). *S. aureus* (ATCC 29213) was purchased by ATCC materials resource. Human immortalized keratinocytes (HaCaT cell line) (CLS Cell Lines Service, 300493) were used for the study. All solutions used for in vivo infusions were prepared using non-pyrogenic saline (0.9% wt/vol NaCl; Baxter Healthcare Ltd., Thetford, United Kingdom). The topical cream containing xyloglucan, pea protein and *Opuntia ficus-indica* extract was kindly provided by DEVINTEC Sagl (Lugano (CH)). DXM was purchased by Teofarma (PV Italy). IMQ cream was commercially available as Aldara^®^ (5% cream, Meda). All other products used for histological analysis were purchased from Bio Optica (Milan, Italy). 

### 4.4. Statistical Evaluation

All values are displayed as mean ± standard error of the mean (SEM) of N observations, where N represents the number of animals studied. Histological samples shown are representative of at least three experiments (histological coloration) performed on different experimental days on the tissue sections collected from all the animals in each group. The results were analyzed by one-way ANOVA followed by a Bonferroni post hoc test for multiple comparisons. A *p*-value of less than 0.05 was considered significant.

## Data Availability

All data of this study are available upon request at the address of the corresponding author.

## References

[1] MacDonald A., Burden A.D. (2007). Psoriasis: Advances in pathophysiology and management. Postgrad. Med. J..

[2] Parisi R., Symmons D.P., Griffiths C.E., Ashcroft D.M., Identification and Management of Psoriasis and Associated ComorbidiTy (IMPACT) Project Team (2013). Global epidemiology of psoriasis: A systematic review of incidence and prevalence. J. Investig. Dermatol..

[3] Afonina I.S., Van Nuffel E., Beyaert R. (2021). Immune responses and therapeutic options in psoriasis. Cell. Mol. Life Sci..

[4] Roberson E.D., Bowcock A.M. (2010). Psoriasis genetics: Breaking the barrier. Trends Genet..

[5] Jiang S., Hinchliffe T.E., Wu T. (2015). Biomarkers of An Autoimmune Skin Disease—Psoriasis. Genom. Proteom. Bioinform..

[6] Zhang B., Liu N., Hao M., Zhou J., Xie Y., He Z. (2021). Plant-Derived Polysaccharides Regulated Immune Status, Gut Health and Microbiota of Broilers: A Review. Front. Vet. Sci..

[7] Owczarczyk-Saczonek A., Czerwińska J., Wygonowska E., Kasprowicz-Furmańczyk M., Placek W. (2021). D-chiro-inositol as a treatment in plaque psoriasis: A randomized placebo-controlled clinical trial. Dermatol. Ther..

[8] Oulahal N., Degraeve P. (2021). Phenolic-Rich Plant Extracts With Antimicrobial Activity: An Alternative to Food Preservatives and Biocides?. Front. Microbiol..

[9] Eddin L.B., Jha N.K., Goyal S.N., Agrawal Y.O., Subramanya S.B., Bastaki S.M.A., Ojha S. (2022). Health Benefits, Pharmacological Effects, Molecular Mechanisms, and Therapeutic Potential of alpha-Bisabolol. Nutrients.

[10] Aengwanich W., Suttajit M. (2013). Effect of polyphenols extracted from tamarind (*Tamarindus indica* L.) seed coat on pathophysiological changes and red blood cell glutathione peroxidase activity in heat-stressed broilers. Int. J. Biometeorol..

[11] Pique N., Gomez-Guillen M.D.C., Montero M.P. (2018). Xyloglucan, a Plant Polymer with Barrier Protective Properties over the Mucous Membranes: An Overview. Int. J. Mol. Sci..

[12] Ajovalasit A., Sabatino M.A., Todaro S., Alessi S., Giacomazza D., Picone P., Di Carlo M., Dispenza C. (2018). Xyloglucan-based hydrogel films for wound dressing: Structure-property relationships. Carbohydr. Polym..

[13] Campolo M., Casili G., Paterniti I., Filippone A., Lanza M., Ardizzone A., Scuderi S.A., Cuzzocrea S., Esposito E. (2020). Effect of a Product Containing Xyloglucan and Pea Protein on a Murine Model of Atopic Dermatitis. Int. J. Mol. Sci..

[14] Esposito E., Campolo M., Casili G., Lanza M., Franco D., Fazio E., Filippone A., Paterniti I., Cuzzocrea S. (2020). Efficacy of Xyloglucan against Escherichia coli Extraintestinal Urinary Tract Infection: An in vivo Study. Microb. Physiol..

[15] Utrilla M.P., Peinado M.J., Ruiz R., Rodriguez-Nogales A., Algieri F., Rodriguez-Cabezas M.E., Clemente A., Galvez J., Rubio L.A. (2015). Pea (*Pisum sativum* L.) seed albumin extracts show anti-inflammatory effect in the DSS model of mouse colitis. Mol. Nutr. Food Res..

[16] Di Lorenzo F., Silipo A., Molinaro A., Parrilli M., Schiraldi C., D’Agostino A., Izzo E., Rizza L., Bonina A., Bonina F. (2017). The polysaccharide and low molecular weight components of Opuntia ficus indica cladodes: Structure and skin repairing properties. Carbohydr. Polym..

[17] Di Domenico E.G., Cavallo I., Bordignon V., Prignano G., Sperduti I., Gurtner A., Trento E., Toma L., Pimpinelli F., Capitanio B. (2018). Inflammatory cytokines and biofilm production sustain Staphylococcus aureus outgrowth and persistence: A pivotal interplay in the pathogenesis of Atopic Dermatitis. Sci. Rep..

[18] Kintarak S., Whawell S.A., Speight P.M., Packer S., Nair S.P. (2004). Internalization of Staphylococcus aureus by human keratinocytes. Infect. Immun..

[19] Srinivasan B., Kolli A.R., Esch M.B., Abaci H.E., Shuler M.L., Hickman J.J. (2015). TEER measurement techniques for in vitro barrier model systems. J. Lab. Autom..

[20] Zhao W., Han L., Bae Y., Manickam D.S. (2019). Lucifer Yellow—A Robust Paracellular Permeability Marker in a Cell Model of the Human Blood-brain Barrier. J. Vis. Exp..

[21] Lowes M.A., Suarez-Farinas M., Krueger J.G. (2014). Immunology of psoriasis. Annu. Rev. Immunol..

[22] Okada K., Matsushima Y., Mizutani K., Yamanaka K. (2020). The Role of Gut Microbiome in Psoriasis: Oral Administration of Staphylococcus aureus and Streptococcus danieliae Exacerbates Skin Inflammation of Imiquimod-Induced Psoriasis-Like Dermatitis. Int. J. Mol. Sci..

[23] Torsekar R., Gautam M.M. (2017). Topical Therapies in Psoriasis. Indian Dermatol. Online J..

[24] Ye L., Lv C., Man G., Song S., Elias P.M., Man M.Q. (2014). Abnormal epidermal barrier recovery in uninvolved skin supports the notion of an epidermal pathogenesis of psoriasis. J. Investig. Dermatol..

[25] Toruniowa B., Jablonska S. (1988). Mast cells in the initial stages of psoriasis. Arch. Dermatol. Res..

[26] Na Takuathung M., Wongnoppavich A., Panthong A., Khonsung P., Chiranthanut N., Soonthornchareonnon N., Sireeratawong S. (2018). Antipsoriatic Effects of Wannachawee Recipe on Imiquimod-Induced Psoriasis-Like Dermatitis in BALB/c Mice. Evid. Based Complement. Altern. Med..

[27] Jabeen M., Boisgard A.S., Danoy A., El Kholti N., Salvi J.P., Boulieu R., Fromy B., Verrier B., Lamrayah M. (2020). Advanced Characterization of Imiquimod-Induced Psoriasis-Like Mouse Model. Pharmaceutics.

[28] Balci D.D., Duran N., Ozer B., Gunesacar R., Onlen Y., Yenin J.Z. (2009). High prevalence of Staphylococcus aureus cultivation and superantigen production in patients with psoriasis. Eur. J. Dermatol..

[29] Afifi T., de Gannes G., Huang C., Zhou Y. (2005). Topical therapies for psoriasis: Evidence-based review. Can. Fam. Physician.

[30] Griffiths C.E.M., Jo S.J., Naldi L., Romiti R., Guevara-Sangines E., Howe T., Pietri G., Gilloteau I., Richardson C., Tian H. (2018). A multidimensional assessment of the burden of psoriasis: Results from a multinational dermatologist and patient survey. Br. J. Dermatol..

[31] de Servi B., Ranzini F., Pique N. (2016). Effect of Utipro((R)) (containing gelatin-xyloglucan) against Escherichia coli invasion of intestinal epithelial cells: Results of an in vitro study. Future Microbiol..

[32] Eutamene H., Beaufrand C., Harkat C., Theodorou V. (2018). The role of mucoprotectants in the management of gastrointestinal disorders. Expert Rev. Gastroenterol. Hepatol..

[33] Fraile B., Alcover J., Royuela M., Rodriguez D., Chaves C., Palacios R., Pique N. (2017). Xyloglucan, hibiscus and propolis for the prevention of urinary tract infections: Results of in vitro studies. Future Microbiol..

[34] Ammar I., Bardaa S., Mzid M., Sahnoun Z., Rebaii T., Attia H., Ennouri M. (2015). Antioxidant, antibacterial and in vivo dermal wound healing effects of Opuntia flower extracts. Int. J. Biol. Macromol..

[35] Park E.H., Chun M.J. (2001). Wound healing activity of *Opuntia ficus-indica*. Fitoterapia.

[36] Elias P.M., Choi E.H. (2005). Interactions among stratum corneum defensive functions. Exp. Dermatol..

[37] Kalia Y.N., Pirot F., Guy R.H. (1996). Homogeneous transport in a heterogeneous membrane: Water diffusion across human stratum corneum in vivo. Biophys. J..

[38] Elias P.M. (2005). Stratum corneum defensive functions: An integrated view. J. Investig. Dermatol..

[39] Cai Y., Fleming C., Yan J. (2012). New insights of T cells in the pathogenesis of psoriasis. Cell. Mol. Immunol..

[40] Danilenko D.M. (2016). An Overview of the Pathogenesis of Immune-mediated Skin Injury. Toxicol. Pathol..

[41] Jiang B.W., Zhang W.J., Wang Y., Tan L.P., Bao Y.L., Song Z.B., Yu C.L., Wang S.Y., Liu L., Li Y.X. (2020). Convallatoxin induces HaCaT cell necroptosis and ameliorates skin lesions in psoriasis-like mouse models. Biomed. Pharmacother..

[42] Lin A.M., Rubin C.J., Khandpur R., Wang J.Y., Riblett M., Yalavarthi S., Villanueva E.C., Shah P., Kaplan M.J., Bruce A.T. (2011). Mast cells and neutrophils release IL-17 through extracellular trap formation in psoriasis. J. Immunol..

[43] Desmet E., Ramadhas A., Lambert J., Van Gele M. (2017). In vitro psoriasis models with focus on reconstructed skin models as promising tools in psoriasis research. Exp. Biol. Med..

[44] Nishihara Y., Kajiura T., Yokota K., Kobayashi H., Okubo T. (2012). Evaluation with a focus on both the antimicrobial efficacy and cumulative skin irritation potential of chlorhexidine gluconate alcohol-containing preoperative skin preparations. Am. J. Infect. Control.

[45] Visser M.J.E., Kell D.B., Pretorius E. (2019). Bacterial Dysbiosis and Translocation in Psoriasis Vulgaris. Front. Cell. Infect. Microbiol..

[46] Nikam V.N., Monteiro R.C., Dandakeri S., Bhat R.M. (2019). Transepidermal Water Loss in Psoriasis: A Case-control Study. Indian Dermatol. Online J..

[47] Wilson V.G. (2014). Growth and differentiation of HaCaT keratinocytes. Methods Mol. Biol..

[48] Wiegand C., Abel M., Ruth P., Hipler U.C. (2009). HaCaT keratinocytes in co-culture with Staphylococcus aureus can be protected from bacterial damage by polihexanide. Wound Repair Regen..

[49] Bracke S., Desmet E., Guerrero-Aspizua S., Tjabringa S.G., Schalkwijk J., Van Gele M., Carretero M., Lambert J. (2013). Identifying targets for topical RNAi therapeutics in psoriasis: Assessment of a new in vitro psoriasis model. Arch. Dermatol. Res..

[50] Guilloteau K., Paris I., Pedretti N., Boniface K., Juchaux F., Huguier V., Guillet G., Bernard F.X., Lecron J.C., Morel F. (2010). Skin Inflammation Induced by the Synergistic Action of IL-17A, IL-22, Oncostatin M, IL-1{alpha}, and TNF-{alpha} Recapitulates Some Features of Psoriasis. J. Immunol..

[51] Benson K., Cramer S., Galla H.J. (2013). Impedance-based cell monitoring: Barrier properties and beyond. Fluids Barriers CNS.

[52] Gao Y., Li S., Wang J., Luo C., Zhao S., Zheng N. (2017). Modulation of Intestinal Epithelial Permeability in Differentiated Caco-2 Cells Exposed to Aflatoxin M1 and Ochratoxin A Individually or Collectively. Toxins.

[53] Duong Q.V., Kintzing M.L., Kintzing W.E., Abdallah I.M., Brannen A.D., Kaddoumi A. (2019). Plasma Rich in Growth Factors (PRGF) Disrupt the Blood-Brain Barrier Integrity and Elevate Amyloid Pathology in the Brains of 5XFAD Mice. Int. J. Mol. Sci..

[54] Ingrassia I., Leplingard A., Darfeuille-Michaud A. (2005). Lactobacillus casei DN-114 001 inhibits the ability of adherent-invasive Escherichia coli isolated from Crohn’s disease patients to adhere to and to invade intestinal epithelial cells. Appl. Environ. Microbiol..

[55] Filippone A., Consoli G.M.L., Granata G., Casili G., Lanza M., Ardizzone A., Cuzzocrea S., Esposito E., Paterniti I. (2020). Topical Delivery of Curcumin by Choline-Calix [4]arene-Based Nanohydrogel Improves Its Therapeutic Effect on a Psoriasis Mouse Model. Int. J. Mol. Sci..

